# Supplementary material for: Physical Mapping of the Anopheles (Nyssorhynchus) darlingi Genomic Scaffolds
Source: Insects. 2021 Feb 15;12(2):164. doi: 10.3390/insects12020164 (PMC7918962; doi:10.3390/insects12020164)
Supplement: Supplementary file 1 [file insects-12-00164-s001.zip › insects-1058945-ffsup/Supplementary Document 1.pdf]

Pb2r

>DGSJ02D08C.b00   740   0   740 ABI trimmed

GGTTTTCCCAGTCACGACGTTGTTACCGACGGCCAGTGAATTGTAATACGACTCACTATA

GGGCGAATTCGAGCTCGGTACCCGGGGATCCCACCTGTGAAAGCTTAGGATTATTTATGT

GACGCTTGATCCAACGACCACCGTCGTCTTCCGTCTGTGGCCGATATCCCGTCCGATCAC

TCGAAATGCATCATACACGAGAGATGCAAGACATCGACATTTCTGCAAACACCCCCCCTC

GCCTCCCACCTACCACGCATTCCACGCAGCAGTGCCCTTGCTTCGTTAGTGAAGCGGATT

GAAACAGCGCCAGACACACGCCGCATTGCGTTAGTCGTCCCACCCGATGTGGCCACCCAA

TCGCGCTACGCATGCCACTTAACACGGCTGTCGGATGTCGAGCGTTGCAGCTGCTGTTGC

TGTTGCCTTCTGCTCCGGGTTAGATATCCGCTACAGGCCCCCGGGGCACTAGAGATTTGA

TTTTCGCAGCCGAGTAATTCCGTTAGAACGCATTAGCGGGTCTGTGTGCGCTGGTTAGCG

CAACCAAACGCCCGACCTCACTTCGAAACTCCTAGCACTAGCCAGCACTGTTGCTGCTGC

TGCTGCTGCTGCACTCGCGCTCAGTGTATGCGCAGTTGCAACCCATTAACCTATCGATAA

GATGTATGGTAAGCAAAACCGCACTGCTACTGGCGTTTGCCCCGCCAGTATGCGCGCTCG

GTGTAAGGTCGAATCGGCTC

>DGSJ02D08C.g00   742   0   742 ABI trimmed

GCTATGACCATGCCCACGCCAAGCTATTTAGGTGAGACTATACAATACTCAAGCTTGCAT

GCCTGCAGGTCGACTCTAGAGGATCCCACCCACAGCTTCGGTCGCTTGGCTGATGAGGAC

TACAAGATCGTCGCCCACAAGGTTGTCCCGTGGCTGTCCCACCAGCTGCGTGAGGCCGTG

AAGGCTGGAGACAGCATCAAGGTTCAGGTGTACATTCGTTGTCTTGGACACCTTGGACAC

CCGGAAATCCTGAACGTCTTCGAACCCTACCTGGAAGGCAAGATTCCCGTCACCCACTTC

CAGCGCCTGGCCATCATTGTTGCCTTCGACCGTCTGGTGGAGAACTACCCGCGTCTGGCC

CGCTCTGTCCTGTTCAAGGTGTACCAGAACACCGGAGATGCCCATGAGGTCCGTTGCGCC

GCCGTCTACCTGTTGGTCCGCACCAAGCCGCCAGTATACATGCTCCAGCGCATGGCTGAG

CAGACCCACTACGACCCCAGCACCTACGTGCGCGCCGCTGTCAAGACTGCTCTGGAGAGC

GCCTCGGAAGCCGATGAGTTCGATGATGATGATGAGTTCTGGCAGAACGCCCAGGCCGCC

ATCAAGCACCTGAACCCCCGCGACTTCAGCCTCCAGTACTCGGGCACCTACCTGCGTGAC

TTTGCCCTTCAGGAACTCGAGCTGTCCTACCGTCTGTACTTCTCCCAGATTGCCTCTGAC

GATCACTTCGTCCCGAGCGGAT

Pb5r

>DGSJ04D09C.b00   442   0   442 ABI trimmed

TGTTTTGATGATTTCACCGAAGAACCGACACCGGTGCGGTTACTGCGATACCGGATGACG

TGGCAAATTTCATTCCAATTCCTTCTCGCACCCGGTTCCCCCGGGGGGTGGCGGGTGTAA

CCGATAGCGTAATGGCAGCAAGGGTAGCAATGGTATCTCATTACTCCAGCCCTTGGCGTA

CGTACTCCGACAACAAAACGAAACGAAATTAAATGAAATGAATGACGACACCCCACGGGC

AGGGTTTGGTTTGGTTGTGGCGTCATTGAATGGTCTTGCCCCCCCGGGGGGGGGGGGGA

AGAGCATCCCCCTAGGTAGCGACATTCCAAATAAAGCCTCCCTGCCGGGGGTTCAGGGGA

AGCTAATGCATAATGTTTACGCATGCTGGTGCGACAAGAACGACCAGCACAGCAATACAC

GCGCTTTCTGCGACTGCGTGAC

>DGSJ04D09C.g00   768   0   768 ABI trimmed

TGTTGCGCTCGTCCTTATCCCATAGCAACGCAACGCATCACCGAGCGGTGTGTACAGTCC

GGGGAAGGAGGAAATCACAGCATTGCCTCAATCTAACCTCCGAGTGCTAGATTGCCGGGA

CCGTAGTACGGAAGTATTGCTAATGGATCCGCTGAGGTGGAAAATATTGCGGTTTACAGT

AGTTTGCCTCACCACAAACGGCTCGTACGGTGGTGGTCTGACTGAGTAATCGGTCAGCAT

CCGGCCATCGGTCGAGAGAGGGAGAGTGCGATAGAGGGATCAGTGACCAGTGTATAGTGC

ATCAAACATCGAACTGAAGCAGCACAACTGCGGTTGGCTCAGGCTGGTTAAAGTCAGCGT

CAACCTCAAGCCGGACAATCTGTATCGGAATGATAAACGGTCAGCTATCGGGGTGTGTTT

GTCATTCACTCCGTGGTCATCGTAAGGATCTATTCAAACGCAAACTGGACTTGAAACAGT

CAACGCAAGCGATAACTGCTATTGTACATCTAGTGGATTCGTTTAAGGATTAAAATTTAA

AAAGGAAGCCTGGGCCATTGTTGTCAAACCTTTCATCTTAATTAACTCATCATATAATCC

GATTCGATGAAGAGCGATACTCGATATCGAAGCACTTGGTAGCATTTCTTTGTAAACCTG

AGAGTGAAAATCTGCACCAAACCTTCCACAAATTGAACTTTATGGGAATCATTTGGGAAT

ATTATATCTTCAGCATCGCTCAAAGATGCTATAAGACTGCTAATGAAA

Pb7b

>DGSJ01A02C.b02 827 0 827 ABI trimmed

GTTTTCCCAGTCCGACGTTGTAAACGACGGCCAGTGAATTGTAATACGACTCACTATAGG

GCGAATTCGAGCTCGGTACCCGGGGATCCCACCTGGTACAAGGTGAGTGTACTAACCCCC

GGGTCCTGGACCGGGACCCGAACTTGGATGAGTTGTGTTTGGTCGCCATCCACCGACCGA

CCGACAGAACGAAGGAAGGTCGGACACCGGAATCACCACCGGACGCTCGGTGCGAGGCAG

ATAAATATCCCGGTCCCTCTTGTCCTCCCTTCCTCGGGGCGAAGTGGTCCACGGAAGTTG

AAGGAGATTGAGCATTCCTCTGGCCACACTCTGACAAGAAAATGTATCACTCTCGACGAC

GCAGACGACGACCAGGACGAAACCAAAGACCACGACCATCCTCTTTTACCTTTTGCGCCT

CACCGAAACACCTCGATTTACCTCCGCTCTTTATCCGCTTCTGCCACGTTTGTTCCACAG

ATGTTCCCGAACCACCCGGACGTCCGCTGGTCGTAAGCTTCACGTCACGTTCGGTCGATT

TATCCTGGGCTCACTCGCAGGACCCCCGAAATGCACCGGTTACAAATTTCATCATCGAAA

CACGGTAAGCCACACCGACCCGGCCCGGCCCGATCCGACCCAACCGGAGCTGTCCGGAGC

AGCCATTTCGTCGGCATTATGTTTAGATTTACACCCTCCAGGTTCAGAATCCATCCAGTT

CGGACCACGGCGGTTGCAGTGGCTGAGGCTGCGACGTATCTGCGCCAACACACACACAGC

CTCTCTCTCTCCCTCTAGAACTCGAATCCCGCTAGAACCGAACCGGG

>DGSJ01A02C.g00 803 0 803 ABI trimmed

ACGCCAAGCTATTTAGGTGAGACTATACAATACTCAAGCTTGCATGCCTGCAGGTCGACT

CTAGAGGATCCCACCGAGCGATGTGACGGATATTGCTAATGGGTATGGAGCGATGACGAG

AGGGCTAGGAGGAGGATGTTGGTCGCATCAGGGATTACGGACGGGGATGGAAGGTGAAAA

CTACTAGATTTATTCCAGGGTGTTGTAATCGTATTTTTAATGCTCTCAAGCCTGTCAAGT

GCGCCATGTTAGTGCTATTTGTTTGTCGAGTGCCGCCAGGGTTAAGGTTTTATTTATAAA

AATACGATTTGTAACGATTTAGAAGGGACTTTTCGATTCATACGGGATTCTTTGCTTTTC

AATGATGAATTTTCCTCAAGGAAAGTGCTACTTTATCCATGGGATATTCGTTCTTGGGGT

TTACTTGTCCATTTACATTCGTTAACAACATTTAAGAAGTGCAGAATTAACTAATCATAG

TAACATCAAAAATTATGCGTGCCAATGTGATTTAGTAATTTTCCAATGTATGATCTTCGT

GAAAATGATTTATTAAAAATATTTGTGAAATAATATTCACAAATCTGTAAATACAAACTA

TTTGAGAATCTTTGGGTATTTAGGAAGATCATACTAGACCGCCACTACCTCAACATGTCG

CTTGAACTCCTTTGGCTTTTAACATCCTTTGGCTTACTTTTATAATTTATATCAAATTTA

TCTTCAATATCGTTTCGTCTCCTTCCTTCTAGGCCTTGCAGGAGTTAAAAACATCTTTGC

AAATCGACCTAAGATCGAGCGAT

Pb17r

>DGSJ01A09C.b02   747   0   747 ABI trimmed

GGGTTTTCCCGTCACGACGTTGTAATACCACGGCCAGTGAATTGTAATACGACTCACTCA

TAGGGCGAATTCGAGCTCGGTACCCGGGGATCCCACGTGCATCCGAAGATCAGCAGTTCA

ACCTGTTGATAGTACGTACTAAGCTCTCATGTTTCACGTACTAAGCTCTCATGTTTAACG

TACTAAGCTCTCATGTTTAACGAACTAAACCCTCATGGCTAACGTACTAAGCTCTCATGG

CTAACGTACTAAGCTCTCATGTTTCACGTACTAAGCTCTCATGTTTGAACAATAAAATTA

ATATAAATCAGCAACTTAAATAGCCTCTAAGGTTTTAAGTTTTATAAGAAAAAAAAGAAT

ATATAAGGCTTTTAAAGCTTTTAAGGTTTCACCGGTGACGATGACGAGATCGAGCATACC

GATGCCACGGTAGTCGTTGCAGGCCATCCCGCAAACGAACTCGTTCATTTTCATCTTGAA

CCACACGTCCCCGGTGCGAGGATCCCGCACATCCATCTTGCCGCTCTTCCACCCGATGAT

CATCTGCTTACTATCGCATCCGAGGATATCGTACGTGGCCATAGCGGTGGCACGGGTTTT

CGATTTAATGCGCCATAGGCGCACACTTTCCTCGAACACACCGATCGTACCATTCTTTAC

GCTGTACATAAACTGACCTGGACGTAGGGTGGCTAAATTTTGGATCTCATTTCCCTCGGC

AAACTCGTGCAGCAGGGTTTCCCTCTT

>DGSJ01A09C.g00   682   0   682 ABI trimmed

GCCAAGCTATTTAGGTGAGACTATAGCATACTCAAGCTTGCATGCCTGCAGGTCGACTCT

AGAGGATCCCACATTACGAAGATCGGAAGCGAGCATTACGGTGCCCCGGTCAACGAGGCC

GACAAGGTGAACATGCTGGTCAACAAGATCGTCCAATCGTTCAACGGTAACTTTGATGAG

CTGGAGCAGATACTGAAGGAACGCAAGAACCTGACGCTGGCCAGCAACGGAGCCGCCGAT

ACGCCGATCGCGGTAGGCGCTGGCGTTTCGCCATCCAGCAGCAGCAGCAGTAGTACAACC

GAGAGCAATGCGTTAACTAGCAGCACCACTGCTAAGCCCAAGAAGAAGAAGAAGCGCCCC

AGCCGTAAGCCGGTCAATGGAACGACTAGTGGCACGAAGAAACCAACGAGCGCCACCGCT

AAACCGACCGTACTGGCCGATACACCGGTCAGTGGAAACGTCACAAAGCCAACGGCACGT

CCATCCAAGAAGCCCACCAAAGTGCGTCGTCCAAGCAACTCCACGGCGAAACCATCGTCG

AAACCCACGACCAAACCGACCAGTGTCAGTTCGGTCAACAAACCGGCACCGGGCGCTGCC

AACGATAGTCCGGCTAAACCGGTGAAACCATCGACCAAACCGAAACCGACGGCTTCCTCG

TCGTCATCGGTGGGCGCTTCCT

Pb18b

>DGSJ02B03C.b00 815 0 815 ABI trimmed

GGTTTTCCAGTCACGACGTTGTTACCGACGGCCAGTGAATTGTAATACGACTCACTATAG

GGCGAATTCGAGCTCGGTACCCGGGGATCCCACGCAGCCCACCCAGACTGAGGGGGATGC

GTGGATGAAGTTTTCCGTTTTTCTCCAGGGAAGATCGAATTGCTTCTTCACACAAAAGGA

TTAATAAAAACATAATAATCGGTGTTTTTTGGAGGTTATTGTGCAGCTTAATGTGACACC

GATTGGTACAATACATCTCACTTGTTTTTACGATGGGAAAACATGACTTTTTGGTACCAC

AAAGTGGGTTTGTTTGGTTGATCCAGCAAGATGATGATGCGCAAGCCATGTTCAAAGTAT

TATAAACATGTTTTTTTCATGTTTTCTTTATTGAAATACTATCCTTTGTACCAACACGAC

GATTCATCTTACAGGGATGCAGACAATTAGCTGTCAAAATTTATTATAGTCCTTTATGAC

CGTATGGCTCAAATGTAAACCTAGTCAAAGCGAAATTAAATGGTTTAATGCGCTCAATTT

GATGCGCTGTTTATGTAACAACGAAACAAGTTGTTTTCTGTCACGTATACTCTCTAGGCA

CTTGAACATAGAAAAACACATTCTGTCCAGTAATGTAAGGCTTCTAATAAGCAATTCGTG

GAATTTGTCTATTAAACTTAAATTGTTTTGTTGAAAAGAAATTGCACGACAAAAAGCGAC

GAGTATGTTTCGTAGAACTACAAAAGAAACACGATTGCAGATAAAAAAAAGTAACGAAAA

ACATTTCCTTTATGCTAATGATAAATACGACTAGC

>DGSJ02B03C.g00 772 0 772 ABI trimmed

GCTATGACCATGACTACGCCAAGCTATTTAGGTGAGACTATACAATACTCAAGCTTGCAT

GCCTGCAGGTCGACTCTAGAGGATCCCACCAAAACATCCGCCACCAGTTCGGGATTGTGT

TCGCTCAGATCCAATCGATTCTTGGTCATTTCTGCTCAAATCAGTGTTCTGATAGTTGAT

CAAATTGATTGCTACCGGAACTGTCCATTTTTATAAAGATCTATCAGAGATCATTGAATA

ATTTGTACATTTTCAATCATTCATTTCGATAGTTGAGGTGCTATATGGATACTTGCTTGC

ATGAATGCATACTGACGCAGATCGCTGGCAGGTCAGTTGCTATCCATATGTGAATCCTAA

TTCCAAGTTGTGTGGAACTGTGTTTTTTGTCGATAGAAATAAGGATGCTGTTATTTACAA

GAAGCAAAAAAACCTAAAGTATCACGTCATCCTGAACCAACGTCATGTATTGGAGCATGG

CCAATGGGTGCCATCTATTAACGTCTTAACGATCACCGTTTGCAATCGCATCCCGAGTGG

TCTAGTAGTCGTTGGTCGGAAACCGGGACATCGGATGGGGACTTCGTGGTCGTGGTATTG

CTGTTTGCCAACGCGTAACTATCGCTTTCTTCTAGCTGGGGCCACAAGGGCTAGTGTACT

TGCACAGGCGCATCGCCATCGTTTGCCCGCCGGCCAACGCCACGGTTCTAATCGGATGAT

ATAATTGTTGCTTTCTCGCTTGTTTGTTCAACGGACTGCTTCGTCGGATCGC

Pb19r

>DGSJ02B06C.b00 829 0 829 ABI trimmed

GGTTTTCCAGTCCGACGTTGTATCCGACGGCCAGTGAATTGTAATACGACTCACTATAGG

GCGAATTCGAGCTCGGTACCCGGGGATCCCACTGTTTTCGGTGGCAATCACTCGGAAGTG

AAGGCGCGTAATTGCATTTCCTTCCTCGCAAGAGAGAGAGAGAGCCTCACGACGACGGTT

AATTACGGCCAAGACGTAAACATCCGCTCGTGCGATATGCTGCTGCCGCTGCTGCCTCTG

CAGCCTTTGAAAGGGAAAGCCGTCGAGATGCCGGCACCCAGCTGGAGTTTTGAAGAGATT

CGTGCAGAAGTCTCGTGTCTGGTGTCTGGTGGGATGCAAAGCACGAAAGAATTCGGAATC

ATTCATCACTCCAACCAGGCGCGCAGGGGATCCACAGACCAGCGCATGAATCATTGAGCA

CCGTGCAACAGCGTGCGGCTAAAAGATGGATTGATGTGTTTGGCCCCCAGGGTGAAAGAG

GTAGCTGCTGTGCACTGCGTGGCCATGCCAAAGGCGGCCAAAGATCAGAGTCATGTTTTT

CAACGCTGCGTCATCACAACGCAGTAGCATGGTGGCATCGTGCTGCTGCGTTCTTGAAAG

TATTTTTGCTCAAGTTGCGGCGTGTGAAGAGAAAATTAAAAACGGAACAACGTATCGCTC

CGCTCGCCAGCCATCAGTCGTTGCGCCGTTAATTGGAGGGCACGTGAAACCAATGGCACA

CAACAGACCCTTTGCTCAGTTCTCGGTACCAGAGGTGCTGTTTTAAATAGCCTCTTCGTC

GCGGTCCATCATCATCAGCAACACACAAGCTGCTTCACACTCTTCTCCC

>DGSJ02B06C.g00 781 0 781 ABI trimmed

GCCAAGCTATTTAGGTGAGACTATACCATACTCAAGCTTGCATGCCTGCAGGTCGACTCT

AGAGGATCCCACAGTTCCGTGGTTTTTGTGATGAGTTTGAGAACGGATAAGAGGTTTGCA

GACCTCGGCCTCACGGCGTGGATCACACGGCAGACGGAGAAATTAGGTAAGTAAACGGGT

TGATCGCTTTTCCAACACCGGCATGAACGATTCTCTCCTTCTAACTTGCAGGACTCCGTC

GACCGACTCCGATTCAGGTGGAGTGTATTCCACGAATATTGCAGGGCCAGGATTGCATCG

GGGCAGCCAAGACCGGATCAGGCAAAACGTTCGCCTTTGCGTTACCGATTCTGCAGAAGC

TGAGCGAAGAACCGACCGCCAACTTTGCGCTGGTGCTTACGCCGACTCACGAGTTGGCCC

ATCAGATCGCGGAACAGTTTATCGTAGCGGGACAACCGATGAATGCGCGCGTTTGTGTTG

TCACAGGAGGAACGGACCAGTTGCTCGAAGCACAGAAATTACAGAGCAGACCACACATTA

TCGTGGCCATGCCGGGTCGATTGGCGGACCATTTGAATGGCTGCAACACGTATTCTTTTG

CCGCCCTCCAGTTTCTCGTCGTGGACGAAGCAGATCGAGTGCTCAGTGGAAGCTTCGATG

ATGATCTAAGAGTAATCGATCGGTTCTTGCCAGCGAAACGACAGAATCTTTTCTTCTCGG

CCACGATGAAGGATTTCCTCAAAACTTCGATCGTGTTCCCGATCGCTCAGGACGTGTTCG

A

Pb20b

>DGSJ01C04C.b02 823 0 823 ABI trimmed

GTTTTCCAGTCCGACGTTGTATCCGACGGCCAGTGAATTGTAATACGACTCACTATAGGG

CGAATTCGAGCTCGGTACCCGGGGATCCCACATCAATAGACTCGCTCCTCGATCAACACG

ACAGCCCCCGGAGGGCCGGAATTTTAATTTCAATTCCTTCGTCGAAAATTCGACTGCAAG

CTACAGAAAGGGGTTAGATTGCTCCCTTTCCTTTCCACCCCTCAGCCCATCCCCGGTCGG

TCGTGATCAGCTCGATCCATCATCACCTCCCGAAAGGCCGCGATAAAGGACCGATTTCAT

TGTTTTAAAGCCCCGGGCGTGGTGGAAGATGAACCGTTTAGCCGTCAATTAAAAGGCAGG

GGGTGCCGAACGCTCCCAAAAAAAAGGATCGTTTCGTGGCCGCTTTATGCCGTTACGCGG

CAAGAGCGAAAACACCGAATGACACAGAACAGTCAGCGGAGCAATTAAAGGCTCAAAACT

CGAGGTCGGCGAAGCCAGCCAGAACAATGTAGCAAAAGGACTCCTTCGGGGCGGGATCGG

TACGGGGGGATAATGAAGCGATAAAAATATTTGACTCATGTTCTCTAATTTTCCAACTAT

CTATACTATGGGCCACACACACACACACACACATGCGCGTGCACGGGATGCCTAGGGCGA

CAAGCGAGCTAGCCGTCGGCTGCAGTGAGGTCCTGCCAAAGAGAATGTGAAGCTCGAGAG

AGAGAGAGGGATTTAATTAGAGCAACCCTTGATAGGCCATTAGGCCAGGAATTTGATTTT

GGGCCGTTTTTCCATCGTCATCGATCGTTGCCCGCCCCCGGAC

>DGSJ01C04C.g00 692 0 692 ABI trimmed

TACGCCAAGCTATTTAAGTGAGACTATACAATACTCAAGCTTGCATGCCTGCAGGTCGAC

TCTAGAGGATCCCACATTAGACAAGGCAGGGTCTGCAATCCCTTCTCAGGAGTACAGCCG

AGGAGTAGCAGGAGAGCCCGGAAAGATGTAGCAGTTTCCAATTTAAGTGATGCGAAAAGA

TAATGAACCCCTTTTGCCCGGATCGGTTTCCCGAAGTAGGTTTTCTTATCTCGTATTCCC

CCCTCAGGAACTACGGATACCACTCTAGGAGGACGGCATGAGATCGGGTTCAGGGAGAAG

CCGGGCGCACAATACAGGTAGCGCTCAGCGCCGCTACAGACATGACAGAGCATAAGCGAA

AAGAGAGAAAGAGAGAGAGAGCGAAAGGGAGGACAAGCTCACAAGGGGTTGTTATCAGTA

TGTTCGCTTTATTCTCGACTATTTTTTTCCCTCGCAATAGGATCAAATCACACAAACACA

GCACACATACGCACAGGCGCGCCGAGCAGAGTGGCGGCGGAGTAATGGAGTGATAGTTGG

TTGGCTAGTTGGTTAATGGTTGGCTGGCCCCGGAGGAGTTGTGATTGACTGGTCGCGCGC

GCCCGTATCTGACAGTTCAATTAAGCGCCCGAAATCTACAGATCCGGCCACCGCTAGGAT

CCCGGCTAGGGTAGGGTAGGGTAGGGTAGTGT

Pb22b

>DGSJ01E05C.b02 582 0 582 ABI trimmed

GGGCGAATTCGAGCTCGGTACCCGGGGATCAGCACCAACGCAACACCCCTCTCTTTTCTC

GCAAGCATGTGTTACAAGATTTATGACTCAGCCTGCACTTGGAGACATCGAGGCTACTCA

AGAAACCATCTTGCAGGCTGCCATTTTCTCTATTCTCTCGCCATTAGGCAATCAAGCATA

AAGCAAATGTTAGTGGAAACACAGCAAGGTAAGGGTTAACTCGAATTTGGTTAATAAAAA

GCACTATAACTGGGCGCGATGCTGTATTGCATTCTGTCACACCCTCTCTCTTTCACACAC

ACACACGCACACACACACACACACACACGCACACACACGGCCGGTGCAGTAATACGGTTA

TCAAATGAAAACAGTTTGTAGCATCACACCGTTAGAGGTACCTCAATTATGTTTTTGTTG

GTCAGCGACTCGTGCGGCTCGTTGTACTCGGTGTACTTCAGCAGCACCTTGTCCATGTCT

GTGCTGGCATACTGATAGAGCTTATTACTGCTGCTAAAGATAATGAGTGCAATTTCACAA

TCACAAAGTACGGACAGTTCGTACGCCTTCTTCATAACTCCA

>DGSJ01E05C.g00 616 0 616 ABI trimmed

CAAGCTATTTAGGTGAGACTATAGCATACTCAAGCTTGCATGCCTGCAGGTCGACTCTAG

AGGATCCCACTTTGATTCTGACTCCGTGCATGACGACCTGCATGAACGTGGATGCCACCC

TTTACGCGCTGGAGGTTTTGAAGAATTACAAAAGATCGCAGAAAATTGGCCGTTTCAAGA

ACCACGAAGCTGTACGTAAACATCTTTTAAAAATTATGTGCTGGGATTATTCACAAGAAT

AGTGGATTTAATTTGTAGGACGTGAGTTTCGAGAAGAGGAGCGGCAGTTATAAACAAACC

CTTTTTGCGGCCGCGGGGAGGGACCACGTTATTTTTGTTGCACGAATTATTGTTGCGGTC

GTCCAGAAATGAGCAAAAATCCCTACCGTGTAGCCGTGTCCGGGTGAGTTTCGGTCCGTT

TTGCGGTAAGCAAAGGGTTGATCGTGCAAGGAGCGAGGAGTGCGAGGCGGGATTGCCGCG

GAATCACGATCCGTGGATGATCAACATCTTGCGAGCGAGATAAAGTGAGCCAGTGCTATG

AAAGATAGAAAGAGCGAGCAATAGTTGTGCAAGGTGGATCGAGCGGAGCGGAGGAATACG

CGAAAGCTTCTCAGAA

Pb23r

>DGSJ01C06C.b02 775 0 775 ABI trimmed

GTTTTCCAGTCCGACGTTGTTTCCGACGGCCAGTGAATTGTAATACGACTCACTATAGGG

CGAATTCGAGCTCGGTACCCGGGGATCCCACTTCTTATTCGGCCTGACGAAATCGAATAT

TTAACGCCACAATTAATTTCAACAATTAAATGCCCCAATTCTCTGTCCTCTCGACCATAC

GATCCAGAAATCCCCGCCCCGCTATCAATCAATCTAGACGACTAATACGACAAGCATATT

CGGTTGTTGTCTTATCGATTGAGCTTTAAAATCCGGCTTTTGAACGAAACGAACGTGTCC

GATAAGGCAATCCGATATAGCGGCCCTCGGACGTTGAAGCACTACAGACAACGCGTGTTA

TCTTTCGCTATCAGCATTCCCACCACCCATCTTATGGTTCAGACATTCGATTGCCAGTCT

GATTCATGGATATGGTACGCGTCGAGGAATGAAATGCCTTCCCGTATCATAAATCAAATC

ACAACGAGCTACGGTTTCTGGGTCACATTTCTCTCTATTTGCTAGTCTTCTTCCGCCAGA

GACTTTCGTTAAAACACTAGGTGCAAAAACACAAATAAGAATTCCACTTACAACGAATCA

AAGTCAATACAAAATGTTCCAGATTCTTCGTTCTCGGGGTAGTGTGTTTGGTAGGCTAAT

GGTAAAGTGGATGAGATTTAACTTGTTCGCGGCCACCGCTCTGCACAAACGGCAAAATCC

TTGGGTAAATACTTCTCCAGGTGCTCTAGCTTCTCGCAACATCATTTCAGTTCGG

>DGSJ01C06C.g00 790 0 790 ABI trimmed

CGCCAAGCTATTTAGGTGAGACTATACAATACTCAAGCTTGCATGCCTGCAGGTCGACTC

TAGAGGATCCCACGTATGATATTTTATTTAATTCATCTCAGCGTTTCGTTGGTCTGCTTT

ATCTAACACATTCGCATTCCAGCGCGGTACTTGTTGCACAGGATGCTGGATGTCCAGCGA

ATGGTAGGACAGTTGTCGAATACCCCCAGGAACATGCAATTATCTATTATCCTTCCCAAA

GCAACGCAGGAGCCAGCACGATTTTTAATATGCTCAATGTTTTGCTTCACATAAACAATA

CAAAGTTCAGCCGCCCGCTTTACAGCAGCTCGATAATCCGCTTCCGGAAACTGGTCGGCC

AGGGCATGTAGTTTAACTTCGTTCAACTCCAAACCCTGGATGATTCCCATCTCTTCATAA

ACACACTGTGATAGACACTGCAACGAATACATTCATCTTCTGAGGTTATTAAAATCATTA

CACCAGTCGAGACTCCAAACCGTACCATTCCGTACTCAATCGATCCAGGATTCTCCTTTG

CAGCCGCTTTCTCCTGACAAACAGGCTCCAATGAGCGTGGAATAGGGTCCTCCAGTCGAC

AGCAGTCCATCAATTCGTGCACCTACAATACATACAACATATTTCAACATAGCGATAACA

CTTCCTTCTTCGAAGAATAGCTGATAATACCTACATCATTACGTGATATGCAGTTGCTAT

CCGCCAGTAGCTAATACCCGTTGCAGGAGGAAAACAGAAAAATATTTTATTAAGAATATA

TTGACGCTAT
