# Supplementary material for: Physical Mapping of the Anopheles (Nyssorhynchus) darlingi Genomic Scaffolds
Source: Insects. 2021 Feb 15;12(2):164. doi: 10.3390/insects12020164 (PMC7918962; doi:10.3390/insects12020164)
Supplement: Supplementary file 1 [file insects-12-00164-s001.zip › insects-1058945-ffsup/Supplementary Table 1.pdf]

Probe	BLAST hits	query	length bp	e - value	score	% identity	query start	query end	hit start	hit end	pobe length (bp)	In situ chromosome mapping *Anopheles darlingi*	In silico chromosome mapping *Anopheles albimanus*	In silico chromosome mapping *Anopheles gambiae*
Pb2r												2R (8A, 10A, 10E)	2R (10A)	2R (18B)
>DGSJ02D08C.b00	NO HIT	DGSJ02D08C.b00												
>DGSJ02D08C.g00	scaffold_732	DGSJ02D08C.g00	653	0	1280	99.2%	90	742	49731	50383	???			
	scaffold_1942	DGSJ02D08C.g00	653	0	1276	99.1%	90	742	4364	5016				
	scaffold_1482	DGSJ02D08C.g00	653	0	1276	99.1%	90	742	2322	2974				
Pb5r												2R (15 B), 3R (31A)	2R (12C)	2R (12C)
>DGSJ04D09C.b00	scaffold_112	DGSJ04D09C.b00	442	9.00E-173	672	90.7%	1	442	129618	130051	38754			
>DGSJ04D09C.g00	scaffold_112	DGSJ04D09C.g00	768	0	1254	92.4%	1	768	91297	92062				
	scaffold_112:91297-130051													
Pb7b												2R (6A)	2R (10B)	2R (13B)
>DGSJ01A02C.b02	scaffold_281	DGSJ01A02C.b02	735	0	1398	97.5%	93	827	88065	88809	31333			
>DGSJ01A02C.g00	scaffold_281	DGSJ01A02C.g00	729	0	1400	98.5%	75	803	57476	58205				
	scaffold_281:57476-88809													
Probe 17r												2L (25C), 2R(9A)	2L (24A)	3R (32D)
>DGSJ01A09C.g00	scaffold_1409	DGSJ01A09C.g00	612	0.00E+00	1114	96.40%	71	682	18126	18739	???			
>DGSJ01A09C.b02	scaffold_1350	DGSJ01A09C.b02	355	5.00E-173	674	98.0%	393	747	13447	13801				
Pb18b												2L (16B)	2L (17A)	3R (29A)
>DGSJ02B03C.b00	scaffold_683	DGSJ02B03C.b00	689	0	1052	90.4%	94	782	18786	19474	???			
>DGSJ02B03C.g00	scaffold_1062	DGSJ02B03C.g00	685	0	1320	98.7%	88	772	13198	13881				
Pb19r												2L (25C)	3L (45A)	2L (21D)
>DGSJ02B06C.b00	scaffold_17	DGSJ02B06C.b00	737	0	1396	97.2%	93	829	370674	371424	35141			
>DGSJ02B06C.g00	scaffold_17	DGSJ02B06C.g00	709	0	1382	99.0%	73	781	336283	336991				
	scaffold_17:336283-371424													
Pb20b												2L (21D,25B), 3R (27B)	X (1A)	X (5B)
>DGSJ01C04C.b02	NO HIT	DGSJ01C04C.b02												
>DGSJ01C04C.g00	scaffold_958	DGSJ01C04C.g00	617	0	1146	97.3%	76	692	18096	18707	???			
Pb22b												3L (43 C)	3L (45A)	2L (28C)
>DGSJ01E05C.b02	scaffold_17	DGSJ01E05C.b02	547	0	1062	98.4%	36	582	39978	40530	33302			
>DGSJ01E05C.g00	scaffold_17	DGSJ01E05C.g00	552	0	986	94.7%	65	616	72729	73280				
	scaffold_17:39978-73280													
Pb23r												2L (22C)	3L (45A)	2L (23D)
>DGSJ01C06C.b02	scaffold_17	DGSJ01C06C.b02	684	0	1298	97.7%	92	775	473864	474554	38604			
>DGSJ01C06C.g00	scaffold_17	DGSJ01C06C.g00	717	0	1374	98.5%	74	790	435950	436668				
	scaffold_17:435950-474554													
