# Supplementary material for: Physical Mapping of the Anopheles (Nyssorhynchus) darlingi Genomic Scaffolds
Source: Insects. 2021 Feb 15;12(2):164. doi: 10.3390/insects12020164 (PMC7918962; doi:10.3390/insects12020164)
Supplement: Supplementary file 1 [file insects-12-00164-s001.zip › insects-1058945-ffsup/Supplementary Table 2.pdf]

Probe	BLAST Hits	Gene IDs
Pb2r		
>DGSJ02D08C.b00	NO HIT	
>DGSJ02D08C.g00	scaffold_732	ADAC001061
	scaffold_1942	ADAC003002
	scaffold_1482	ADAC001064
Pb5r		
>DGSJ04D09C.b00	scaffold_112	
>DGSJ04D09C.g00	scaffold_112	
	scaffold_112:91297-130051	No annotated gene
Pb7b		
>DGSJ01A02C.b02	scaffold_281	
>DGSJ01A02C.g00	scaffold_281	
	scaffold_281:57476-88809	ADAC000747, ADAC000745, ADAC000750
Probe 17r		
>DGSJ01A09C.g00	scaffold_1409	ADAC001611 (within 5Kb from Blast hit)
>DGSJ01A09C.b02	scaffold_1350	ADAC006083, ADAC006086 (within 5Kb from Blast hit)
Pb18b		
>DGSJ02B03C.b00	scaffold_683	No annotated gene
>DGSJ02B03C.g00	scaffold_1062	No annotated gene
Pb19r		
>DGSJ02B06C.b00	scaffold_17	
>DGSJ02B06C.g00	scaffold_17	
	scaffold_17:336283-371424	ADAC002722, ADAC002737
Pb20b		
>DGSJ01C04C.b02	NO HIT	
>DGSJ01C04C.g00	scaffold_958	ADAC000657 (within 5Kb from Blast hit)
Pb22b		
>DGSJ01E05C.b02	scaffold_17	
>DGSJ01E05C.g00	scaffold_17	
	scaffold_17:39978-73280	ADAC002704, ADAC002694, ADAC002696
Pb23r		
>DGSJ01C06C.b02	scaffold_17	
>DGSJ01C06C.g00	scaffold_17	
	scaffold_17:435950-474554	ADAC002731, ADAC002734, ADAC002703, ADAC002712,<br>ADAC002732, ADAC002738, ADAC002733
