# Supplementary material for: Physical Mapping of the Anopheles (Nyssorhynchus) darlingi Genomic Scaffolds
Source: Insects. 2021 Feb 15;12(2):164. doi: 10.3390/insects12020164 (PMC7918962; doi:10.3390/insects12020164)
Supplement: Supplementary file 1 [file insects-12-00164-s001.zip › insects-1058945-ffsup/Supplementary Table 3.pdf]

Gene ID	Genomic Location (Gene)	Product Description	PFam Description	Smart Description	Computed GO Functions
ADAC000657	ADMH02000163:18,748..20,654(-)	cytochrome P450 4g15 [Source:LNCC;Acc:AD00657]	Cytochrome P450	N/A	heme binding;iron ion binding;metal ion binding;monooxygenase activity;oxidoreductase activity
ADAC000745	ADMH02000186:71,906..94,376(-)	unspecified product	Fibronectin type III	Fibronectin type III;Immunoglobulin subtype	protein binding
ADAC000747	ADMH02000186:67,018..70,655(-)	Protein tyrosine phosphatase 99A [Source:LNCC;Acc:AD00747]	PTP type protein phosphatase	PTP type protein phosphatase;Protein-tyrosine phosphatase, catalytic	hydrolase activity;phosphatase activity;phosphoprotein phosphatase activity;protein tyrosine phos...
ADAC000750	ADMH02000186:61,779..62,105(+)	s-adenosyl-methyl transferase mraw Partial [Source:LNCC;Acc:AD00750]	N/A	N/A	transferase activity
ADAC001061	ADMH02000279:47,573..52,665(+)	vitellogenin-A1 [Source:LNCC;Acc:AD01061]	Lipid transport protein, N-terminal;Vitellinogen, open beta-sheet	Lipid transport protein, N-terminal;Vitellinogen, open beta-sheet	lipid transporter activity
ADAC001064	ADMH02000280:164..6,474(+)	vitellogenin-A1 [Source:LNCC;Acc:AD01064]	von Willebrand factor, type D domain;Lipid transport protein, N-terminal;Vitellinogen, open beta-...	von Willebrand factor, type D domain;Lipid transport protein, N-terminal;Vitellinogen, open beta-...	lipid transporter activity
ADAC001611	ADMH02000416:14,873..17,534(-)	serine protease Partial [Source:LNCC;Acc:AD01611]	Serine proteases, trypsin domain	Serine proteases, trypsin domain	hydrolase activity;peptidase activity;serine-type endopeptidase activity;serine-type peptidase ac...
ADAC001991	ADMH02000489:37,286..41,749(+)	unspecified product	Cyclin, N-terminal	N/A	
ADAC001999	ADMH02000489:35,004..35,573(+)	Transmembrane protein 234 homolog [Source:Projected from Anopheles gambiae (AGAP012180) UniProtKB...	Putative transmembrane family 234	N/A	
ADAC002694	ADMH02000632:47,439..56,535(-)	unspecified product	N/A	N/A	
ADAC002696	ADMH02000632:63,025..71,237(-)	unspecified product	N/A	N/A	
ADAC002703	ADMH02000632:466,687..471,182(+)	molybdopterin cofactor synthesis protein a [Source:LNCC;Acc:AD02703]	Molybdopterin cofactor biosynthesis C (MoaC) domain;Radical SAM	Elp3/MiaB/NifB	4 iron, 4 sulfur cluster binding;catalytic activity;iron-sulfur cluster binding;metal ion binding
ADAC002704	ADMH02000632:36,573..40,687(-)	myocyte-specific enhancer factor 2d [Source:LNCC;Acc:AD02704]	Transcription factor, MADS-box;Holliday junction regulator protein family C-terminal	Transcription factor, MADS-box	DNA binding;RNA polymerase II transcription regulatory region sequence-specific DNA binding;prote...
ADAC002712	ADMH02000632:439,415..442,478(-)	notum [Source:LNCC;Acc:AD02712]	Pectinacetylesterase/NOTUM	N/A	hydrolase activity
ADAC002722	ADMH02000632:336,301..337,999(+)	ATP-dependent RNA helicase DBP8 [Source:LNCC;Acc:AD02722]	DEAD/DEAH box helicase domain;Helicase, C-terminal	Helicase superfamily 1/2, ATP-binding domain;Helicase, C-terminal	ATP binding;helicase activity;hydrolase activity;nucleic acid binding;nucleotide binding
ADAC002731	ADMH02000632:437,284..438,248(+)	odorant-binding protein [Source:Projected from Anopheles gambiae (AGAP006074) VB Community Annota...	N/A	N/A	
ADAC002732	ADMH02000632:471,304..471,918(-)	endothelial precursor protein B9 [Source:LNCC;Acc:AD02732]	B9 domain	N/A	
ADAC002733	ADMH02000632:472,599..473,187(-)	prefoldin [Source:LNCC;Acc:AD02733]	Prefoldin beta-like	N/A	unfolded protein binding
ADAC002734	ADMH02000632:463,803..464,375(+)	unspecified product	N/A	N/A	
ADAC002737	ADMH02000632:338,192..338,584(-)	40S ribosomal protein S24 [Source:LNCC;Acc:AD02737]	Ribosomal protein S24e	N/A	structural constituent of ribosome
ADAC002738	ADMH02000632:473,567..473,872(+)	Protein with signal anchor [Source:UniProtKB/TrEMBL;Acc:W5JN37]	N/A	N/A	
ADAC003002	ADMH02000727:870..7,174(-)	vitellogenin-A1 [Source:LNCC;Acc:AD03002]	von Willebrand factor, type D domain;Lipid transport protein, N-terminal;Vitellinogen, open beta-...	von Willebrand factor, type D domain;Lipid transport protein, N-terminal;Vitellinogen, open beta-...	lipid transporter activity
ADAC006083	ADMH02001516:9,879..14,948(-)	Bardet-Biedl syndrome 2 protein [Source:Projected from Anopheles gambiae (AGAP009637) VB Communit...	Ciliary BBSome complex subunit 2, N-terminal;Ciliary BBSome complex subunit 2, C-terminal domain;...	N/A	
ADAC006086	ADMH02001516:15,980..16,999(+)	CDK-activating kinase assembly factor MAT1 [Source:LNCC;Acc:AD06086]	Cdk-activating kinase assembly factor MAT1, centre;Zinc finger, RING-type	Zinc finger, RING-type	cyclin-dependent protein serine/threonine kinase activator activity;kinase activity;ligase
